# High Prevalence of Both Humoral and Cellular Immunity to *Zaire ebolavirus* among Rural Populations in Gabon

**DOI:** 10.1371/journal.pone.0009126

**Published:** 2010-02-09

**Authors:** Pierre Becquart, Nadia Wauquier, Tanel Mahlakõiv, Dieudonné Nkoghe, Cindy Padilla, Marc Souris, Benjamin Ollomo, Jean-Paul Gonzalez, Xavier De Lamballerie, Mirdad Kazanji, Eric M. Leroy

**Affiliations:** 1 Unité des Maladies Virales Emergentes, Centre International de Recherches Médicales de Franceville, Franceville, Gabon; 2 UMR190 Emergence des Pathologies Virales, Université Aix-Marseille II & Institut de Recherche pour le Développement, Marseille, France; 3 Mahidol University at Salaya, Nakhonpathon, Thailand; U.S. Naval Medical Research Center Detachment/Centers for Disease Control, United States of America

## Abstract

To better understand *Zaire ebolavirus* (ZEBOV) circulation and transmission to humans, we conducted a large serological survey of rural populations in Gabon, a country characterized by both epidemic and non epidemic regions. The survey lasted three years and covered 4,349 individuals from 220 randomly selected villages, representing 10.7% of all villages in Gabon. Using a sensitive and specific ELISA method, we found a ZEBOV-specific IgG seroprevalence of 15.3% overall, the highest ever reported. The seroprevalence rate was significantly higher in forested areas (19.4%) than in other ecosystems, namely grassland (12.4%), savannah (10.5%), and lakeland (2.7%). No other risk factors for seropositivity were found. The specificity of anti-ZEBOV IgG was confirmed by Western blot in 138 individuals, and CD8 T cells from seven IgG+ individuals were shown to produce IFN-γ after ZEBOV stimulation. Together, these findings show that a large fraction of the human population living in forested areas of Gabon has both humoral and cellular immunity to ZEBOV. In the absence of identified risk factors, the high prevalence of “immune” persons suggests a common source of human exposure such as fruits contaminated by bat saliva. These findings provide significant new insights into ZEBOV circulation and human exposure, and raise important questions as to the human pathogenicity of ZEBOV and the existence of natural protective immunization.

## Introduction

*Ebolavirus* (EBOV) and its close relative *marburgvirus* (MARV) compose the *Filoviridae* family of viruses causing severe hemorrhagic fever (HF) in humans and non human primates [1]. The EBOV genome is about 19 000 nucleotides long and consists of a single strand of negative-sense RNA that encodes seven linearly arranged gene products, in the following order: the nucleoprotein (NP), VP35, VP40, glycoprotein, VP30, VP24, and the polymerase (L). The genus *Marburgvirus* consists of a single species, while there are five known species of Ebola-like-viruses that have different geographic locations and case fatality rates, and about 32% to 41% of nucleotide sequence differences [2]. The species *Reston ebolavirus* was first isolated from Asian cynomolgus monkeys from the Philippines; it is pathogenic for non human primates but apparently non pathogenic for humans [3], [4]. Recently, *Reston ebolavirus* was also isolated from domestic Philippino swine with a severe respiratory syndrome and coinfected by porcine reproductive and respiratory syndrome virus [5]. The species *Côte d'Ivoire ebolavirus* has been associated with a single, non fatal human case, in Ivory Coast in 1994 [6]. *Sudan ebolavirus* has caused four known outbreaks, three in Sudan [7]–[9] and one in Uganda [10], [11] with a reported case fatality rate of around 50%. The latest species to be discovered, *Bundibugyo ebolavirus*, was discovered in 2007 in Uganda, where it was responsible for a large outbreak, with 116 confirmed cases and 30 deaths (case fatality rate 26%) [12]. *Zaire ebolavirus* (ZEBOV) is the most pathogenic species, with reported case fatality rates of up to 90%. ZEBOV has caused several outbreaks in Central Africa, Democratic Republic of Congo (DRC), Republic of Congo (RC) and Gabon [13]–[17]. North-east Gabon experienced four outbreaks between 1994 and 2002, with a total of 259 confirmed human cases and only 79 survivors (case fatality rate: 69%).

Recently, significant advances have been made in our understanding of filovirus ecology. Antibodies and nucleotide sequences specific for ZEBOV [18], [19] have been detected in the liver and spleen of three fruit bat species in Gabon and RC (*Hypsignathus monstrosus*, *Epomops franquetti*, and *Myonycteris torquata*), and antibodies and nucleotide sequences specific for MARV [20], [21] have been found in a fruit bat species in Gabon (*Rousettus aegyptiacus*) and in two insectivorous bat species in DRC (*Rhinolophus eloquens* and *Miniopterus inflatus*). More recently, MARV was isolated for the first time in cave-dwelling *Rousettus aegyptiacus* in Uganda [22]. Together, these findings raise the possibility that these bats might be a filovirus reservoir, but the mechanism of primary ZEBOV transmission to humans, potentially leading to outbreaks, remains unclear. The Ebola hemorrhagic fever (EHF) outbreaks that occurred in Gabon and RC between 2001 and 2003 were also associated with major outbreaks among wild-living large mammals (especially chimpanzees and gorillas), devastating local animal populations [16], [23], [24]. The primary human cases involved hunters who became infected after handling animal carcasses found in the forest [16]. Similarly, the 1996 Mayibout outbreak in Gabon started among children who handled a chimpanzee carcass [15]. A recent study showed that the 2007 Luebo outbreak in DRC was linked to massive fruit bat migration, strongly suggesting for the first time that humans could be infected directly by bats [25]. However, the source of most EBOV and MARV outbreaks has not been identified.

It is generally accepted that ZEBOV is associated with a case fatality rate of about 90%, but this may be an overestimate. First, seven cases of asymptomatic infection were identified during the 1996 Booué outbreak in Gabon [26]. Second, some ELISA-based serosurveys [27] have shown high antibody prevalence rates among populations living in areas where no cases of EHF have ever been reported, suggesting that ZEBOV might also be capable of causing mild illness or even asymptomatic infection in humans. The IgG seroprevalence was 9.3% in villages located in the 1995 outbreak area around Kikwit, DRC, where no EHF cases were reported [28]. Likewise, a seroprevalence of 13.2% was found in the Aka Pygmy population of Central African Republic, where no ZEBOV outbreaks have ever been reported [29]. These findings confirmed those of older studies based on less-specific immunofluorescence assays that showed an antibody prevalence of around 10% in several non epidemic parts of Africa [30]–[34]. In contrast, a more recent survey showed a low anti-ZEBOV IgG prevalence (1.4%) among 979 people living in the northern region of Gabon that experienced EHF outbreaks between 1994 and 1997 [35]. The authors deduced that mild or asymptomatic EHF infection was possible but rare.

The source and significance of anti-ZEBOV IgG seropositivity among people who have never had clinical signs of hemorrhagic fever or who live in non epidemic areas are both unclear, but they may have major implications for our understanding of the epidemiology of ZEBOV of primary transmission to humans and outbreaks. We therefore conducted a very large serological survey of ZEBOV exposure among rural populations of Gabon, a country with both epidemic and non epidemic regions. The specificity of ZEBOV-specific IgG was confirmed by western blot, and ZEBOV-specific memory T cell responses were investigated for the first time.

## Results

### Survey Participation

**Figure 1:**
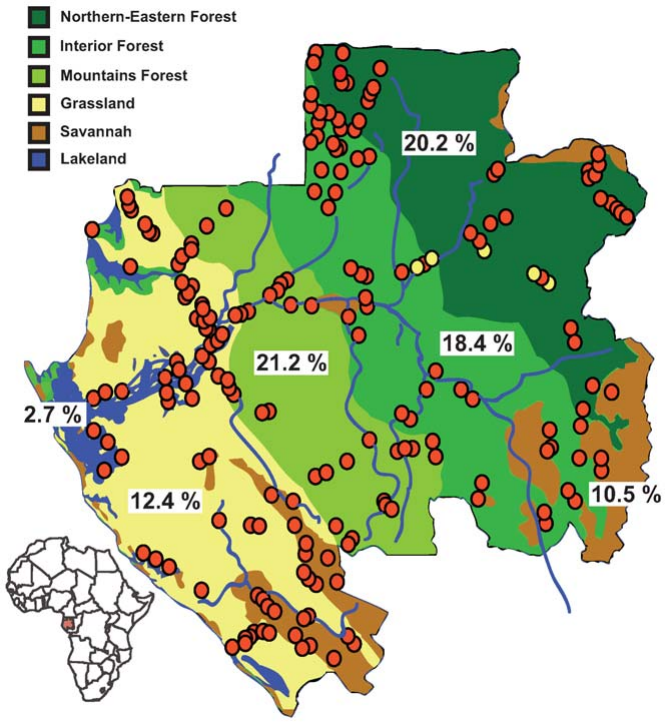
Map of Gabonese villages (red circles) included in the survey, according to the ecological region. Villages where children <16 years were specifically included in the study are indicated by yellow circles. The overall prevalence of ZEBOV-specific IgG in each ecological region is shown.

We enrolled 4,349 individuals in 220 randomly selected villages covering all the ecological regions of the country (Figure 1). Blood samples and sociodemographic data were available for all 4,349 participants. All participants were ≥16 years old, and 2,227 (51.2%) females and 2,122 (48.4%) males participated. The participants were located in the Forest (2,540 participants; 58.4% of the study population), Grassland (918; 21.1%), Savannah (448; 10.3%) and Lakeland (443; 10.2%) regions. The Forest area was subdivided into North-Eastern Forest (825; 19%), Interior Forest (1,314; 30.2%) and Mountain Forest (401; 9.2%).

### Cut-Off Calculation

The cut-off value for IgG positivity was determined by using a negative control population of volunteers sampled in Marseille, France in 2008. Nothing was known of ZEBOV circulation in the area studied. It was therefore impossible to define a control population with which to determine the OD distribution in seronegative subjects: inclusion of seropositive subjects among the controls would have undermined the validity of this distribution and the determination of a valid cutoff. We therefore chose a more rigorous approach, based on the choice of control subjects who were almost certain to be seronegative, yielding a valid “background” OD distribution; we also chose a curve largely covering this distribution and made a conservative choice of cutoff (error risk below 1/1000 for the first “positive” value). The cutoff was therefore based on a highly conservative approach. Finally, the OD distribution of the Gabonese population sample showed a strong excess of values in the range [0.05, 0.20] (relative to the reference distribution), but, thanks to the use of a conservative approach, these values were not considered as positive.

The distribution in the negative control population was centered near 0 (mean 0.006, median 0.009), with a standard deviation of 0.071 (data not shown). This distribution can be surrounded in the positive values by a negative exponential distribution (y = a*exp(-35*(x-0.006))). The exponential distribution gave a better fit than normal and gamma distributions. With this formal distribution, the observed distribution of all ODs was under the curve and the probability of an individual having an OD above 0.2 was less than 1‰. A sample was considered positive when its adjusted OD was above the cut-off (0.2 at 1∶1600 dilution) and when the OD in the viral antigen-coated well was twice as high as the OD in the uninfected antigen-coated well. All samples were tested in duplicate.

### ZEBOV-Specific IgG Seroprevalence and Regional Distribution

The seroprevalence of ZEBOV-specific IgG in the study population was 15.3% overall (Table 1), and varied significantly according to the ecological area (Figure 1, Table 1). The seroprevalence rate in the Forest region (19.4%) was significantly higher than that found in the other regions (p<0.001). Similarly, the seroprevalence rate in the Lakeland region (2.7%) was significantly lower than that found in the other areas (p<0.001). No significant difference was observed (p≥0.3) between Savannah (10.5%) and Grassland (12.4%) nor between the three types of Forest area (p≥0.4) (Table 1).

**Table 1:** Prevalence of ZEBOV-specific IgG in Gabon according to the ecological region.

	N	positive	prevalence	*p* value		
All participants	4,349	667	15.3%			
Lakeland area	443	12	2.7%	<0.001	<0.001	<0.001
Savannah area	448	47	10.5%	<0.001	0.3	referent
Grassland	918	114	12.4%	<0.001	referent	
Forest aera	2,540	494	19.4%	referent		
Northern-Eastern	825	167	20.2%			
Interior	1,314	242	18.4%	0.4		
Mountains	401	85	21.2%			

As shown in Table 2, no significant difference (p≥0.9) in seroprevalence was observed across villages where no ZEBOV outbreaks had been reported (19.5%), villages hit by the 1996–1997 outbreaks (20.2%), and villages hit by the 2001–02 outbreak (18.3%).

**Table 2:** Prevalence of ZEBOV-specific IgG in Gabon according to epidemic and non epidemic areas in Gabon.

Forest area	N	positive	prevalence	*p* value
Villages without outbreak	2,307	449	19.5%	referent
Villages hit by 1996 outbreaks	129	26	20.2%	0.9
Villages hit by 2001 outbreak	104	19	18.3%	0.9

**Figure 2:**
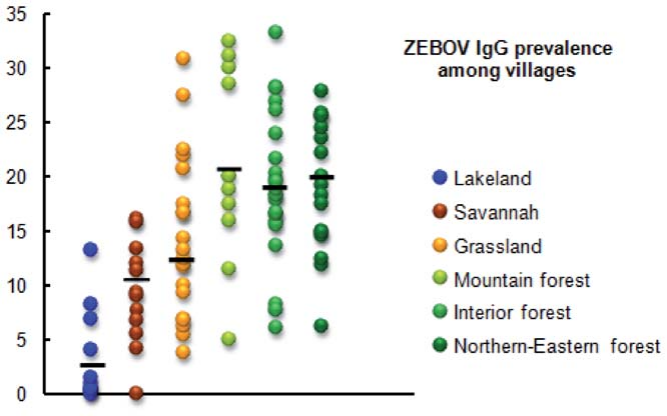
Prevalence of ZEBOV-specific IgG in villages within each ecological region. Each circle represents a group of two or three neighboring villages.

Seroprevalence rates varied widely across neighboring villages in each ecological region (Figure 2), ranging from 0% to 13.2% in Lakeland, 0% to 16.1% in Savannah, 3.8% to 30.8% in Grassland, and 5% to 33.3% in Forest.

### Influence of Sociodemographic Characteristics on ZEBOV-Specific IgG Seroprevalence

Because the seroprevalence rates varied across the ecological areas, we examined the possible influence of sociodemographic characteristics (Table 3). No correlation was found between the seroprevalence rate and gender, age, hunting activity or contact with specific forest animals in a given ecological area (Table 3). The ‘*hunting*’ subgroup consisted of men who hunted frequently or occasionally, and thus excluded females. The ‘*contact with specific forest animals*’ subgroup included hunters, individuals who kept wild animals as pets, those who butchered dead animals, and those who cooked them.

**Table 3:** Prevalence of ZEBOV-specific IgG according to demographic characteristics in individuals aged ≥20 years, Gabon.

	Forest			Grassland			Savannah			Lakeland		
	N	Pos (%)	p value	N	Pos (%)	p value	N	Pos (%)	p value	N	Pos (%)	p value
Male	1,181	248 (21%)	0,07	471	72 (15.3%)	0.01	191	25 (13.1%)	0.1	206	9 (4.4%)	0.09
Female	1,359	246 (18.1%)		447	42 (9.4%)		257	22 (8.6%)		237	3 (1.3%)	
Age (years)												
21–30	343	275 (19.6%)	0.4	122	14 (11.5%)	0.7	55	5 (9.1%)	0.9	70	3 (4.3%)	0.7
31–40	425	343 (19.3%)		157	17 (10.8%)		74	8 (10.8%)		82	3 (3.7%)	
41–50	498	390 (21.7%)		197	29 (14.7%)		102	10 (9.8%)		72	2 (2.8%)	
≥51	1186	215 (18.1%)		404	51 (12.6%)		197	23 (11%)		209	4 (1.9%)	
Hunting	775	163 (21%)	0,9	262	43 (16.4%)	0.5	128	14 (10.9%)	0.3	67	6 (8.9%)	0.07
No hunting	403	85 (21.1%)		209	29 (13.9%)		63	11 (17.5%)		135	3 (2.2%)	
Contact with animals	2473	481 (19.4%)	0,9	ND			ND			ND		
No contact with animals	67	13 (19.4%)		ND			ND			ND		

**Figure 3:**
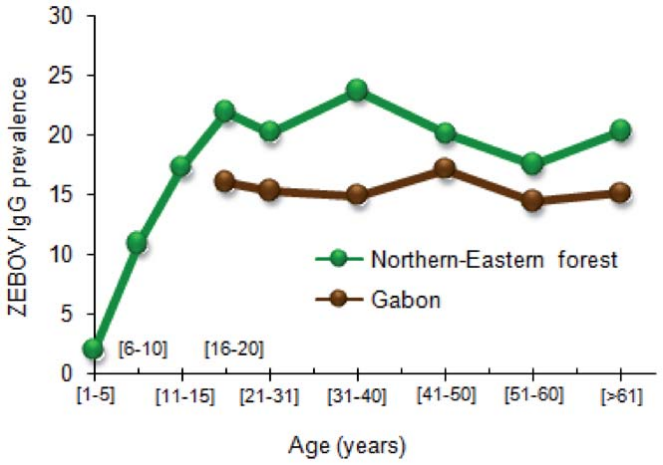
Prevalence of ZEBOV-specific IgG according to age in the north-eastern region of Gabon (green). The overall prevalence of ZEBOV-specific IgG according to age is shown in brown.

The seroprevalence did not significantly vary with age (Table 3). However, only individuals older than 21 years were included in this analysis, owing to the small number of younger participants. In order to evaluate the influence of age among individuals younger than 21 years, we conducted a specific field study of children living in six villages located in the north-east forest area. We enrolled 395 children between 2 and 15 years old. The seroprevalence rate was significantly lower in the 1–10 year age group (8.7%; p = 0.005) than in all the other age groups (Table 4). The seroprevalence rates were 18.5% in the 11–20 year age group, 20.2% in the 21–30 year age group, 23.7% in the 31–40 year age group, 20.1% in the 41–50 year group and 18.5% in the ≥51 year group. The seroprevalence rates increased linearly with age below 15 years (Figure 3).

**Table 4:** Prevalence of ZEBOV-specific IgG according to age in the north-eastern region of Gabon.

	Northern-Eastern forest			
Age (years)	N	Pos (%)	p value	
1–10	207	18 (8.7%)	0.005	
11–20	232	43 (18.5%)		0.7
21–30	124	25 (20.2%)		
31–40	152	36 (23.7%)		
41–50	164	33 (20.1%)		
≥51	341	63 (18.5%)		

### Detection of Specific Anti-ZEBOV IgG by Western Blot

**Figure 4:**
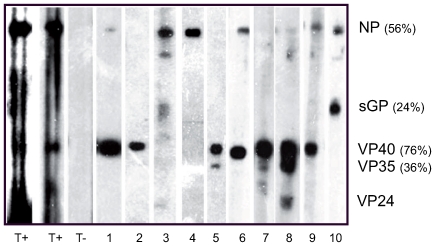
Western blot analysis of ZEBOV-specific IgG from two symptomatic individuals who recovered (T+), one negative endemic control, and 10 IgG+ asymptomatic individuals.

To confirm the specificity of anti-ZEBOV IgG, 150 randomly selected sera of the 667 positive samples were analyzed by western blotting in denaturing conditions with purified ZEBOV antigens, but technical problems meant that clear-cut results could not be obtained for 12 of them. All 138 sera tested by western blot (about 21% of all the positive samples) reacted with at least one ZEBOV protein. These selected sera represented a broad range of ODs and a broad geographic area of Gabon. Depending on the individual, IgG reactivity was directed against NP, VP40, VP35 VP24 and/or sGP (Figure 4). In total, 76% of the 138 sera reacted to VP40, 56% to NP, 36% to VP35 and 24% to sGP, while only one sample reacted to VP24. The IgG reactivity of serum from two survivors was also mainly directed to NP and VP40 (Figure 4).

### ZEBOV-Specific T Lymphocyte Memory Responses

To detect ZEBOV-specific T cell memory responses in these IgG+ individuals, intracellular levels of TNF-α and IFN-γ were determined in CD4+ and CD8+ T cell populations from seven randomly selected IgG+ individuals. Positive gating for lymphocytes based on forward and side scatter was followed by CD3+CD4+ and CD3+CD8+ gating, and specific populations were further defined by using antibodies specific for CD8 and CD4, respectively. Cytokine-positive cells were expressed as a percentage of the corresponding lymphocyte subset.

**Figure 5:**
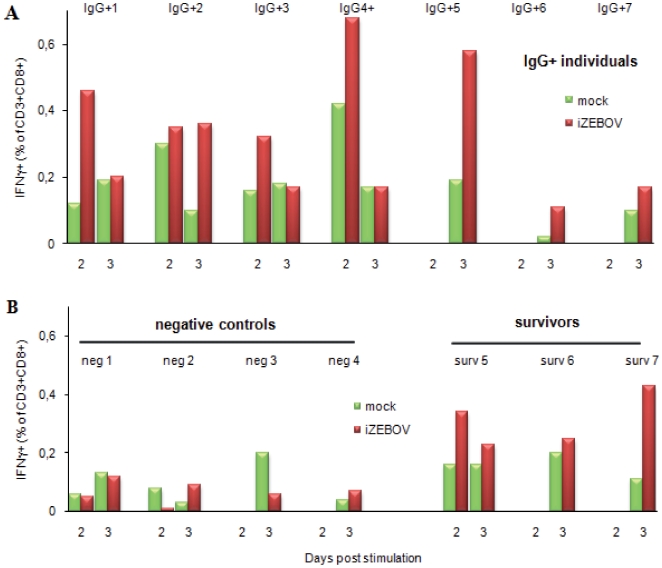
ZEBOV-specific memory T cell analysis by flow cytometry. IFN-γ production by CD3+CD8+ T lymphocytes was evaluated by intracellular flow cytometry on PBMC stimulated with mock supernatant (green histograms) or heat-inactivated ZEBOV culture supernatant (iZEBOV, red histograms). Analysis performed 2 and 3 days after stimulation of PBMC from seven IgG+ asymptomatic individuals (**A**), four negative controls and three laboratory-confirmed survivors of the 2001–2002 outbreak in Gabon (**B**). No significant responses were observed in CD4+ T lymphocytes.

With samples from IgG+ individuals and from three survivors of ZEBOV infection, the percentage of circulating CD4+ T cells producing IFN-γ did not increase after PBMC stimulation (data not shown). In contrast, with samples from the seven IgG+ individuals, the percentage of circulating CD8+ T cells producing IFN-γ was higher on day 2 or 3 of PBMC stimulation by iZEBOV than after mock stimulation (Figure 5). This increase was noted on day 2 in individuals #IgG+1, IgG+3 and IgG+4, and on day 3 in individuals #IgG+2, IgG+5, IgG+6 and IgG+7. The strongest responses were observed in individuals #IgG+1, IgG+2 and IgG+3, with a 3-fold increase. Similar levels of IFN-γ-producing CD8+ T cells were observed in PBMC obtained from three survivors of ZEBOV infection, after iZEBOV stimulation. By contrast, no increase was observed with samples from the four IgG-negative controls (Figure 5).

**Figure 6:**
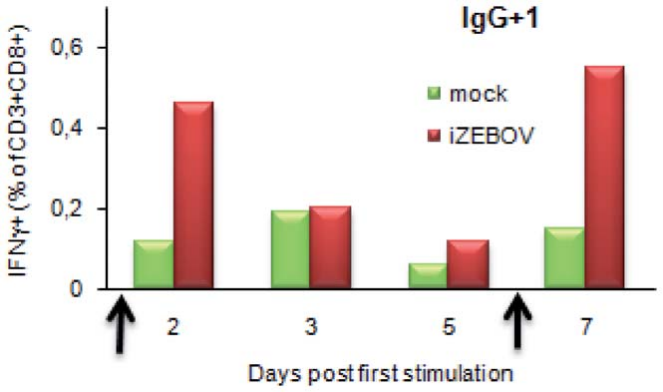
ZEBOV-specific memory T cell analysis by flow cytometry. IFN-γ production by CD8+ T lymphocytes was evaluated by intracellular flow cytometry on PBMC stimulated with mock supernatant (green histograms) or heat-inactivated ZEBOV culture supernatant (iZEBOV, red histograms). Analyses performed 2, 3, 5 and 7 days after initial iZEBOV or mock stimulation (arrow) of PBMC from three IgG+ asymptomatic individuals. A second round of stimulation was performed on day 6. Results for one IgG+ individual are shown.

PBMC from one individual were stimulated with iZEBOV on day 0 and again on day 6. The percentage of circulating IFN-γ-producing CD8+ T rose one day after each stimulation, i.e. on day 2 and day 7 (Figure 6). No increase in the percentage of TNF-α-producing CD8+ or CD4+ T cells was observed with PBMC from IgG+ individuals, although both CD8+ and CD4+ T cells from all three survivors produced low levels of TNF-α on day 3 after iZEBOV stimulation (data not shown).

## Discussion

To our knowledge this is the largest human serological survey of Ebola virus conducted to date. It lasted three years and covered 4,349 individuals in 220 randomly selected villages, representing 10.7% of all villages in Gabon. The overall seroprevalence of ZEBOV-specific IgG was 15.3%, the highest rate ever found. The seroprevalence was even higher in forested areas (representing ¾ of the total surface area of Gabon), reaching 19.4% overall and 33.8% in some villages. The lowest seroprevalence was found in the Lakeland region (2.7%).

These results are consistent with previous studies showing seroprevalence rates ranging from 1.8% to 21.3% [30]–[34]. However, these latter surveys used a poorly specific IFAT method associated with frequent false-positive results [27]. Two recent small serosurveys based on the same ELISA assay as that used here showed elevated seroprevalence rates in some forested regions of central Africa. For example, ZEBOV-specific IgG was found in 9.3% of 161 individuals living in unaffected villages near Kikwit, a few weeks after the 1995 outbreak [28]. Likewise, a rate of 13.2% was found among 190 Aka Pygmies in Central African Republic, where ZEBOV outbreaks have never been reported [29]. However, these surveys involved only small numbers of individuals and a low plasma dilution (1∶400), and the specificity of ZEBOV IgG was not confirmed in more specific tests such as western blot.

We used western blot, for the first time, to confirm the specificity of ZEBOV IgG in 138 ELISA-positive samples selected randomly from various regions of Gabon (about one in five of the positive samples). As previously shown among survivors [36] and in individuals with asymptomatic infection [26], we found that anti-ZEBOV IgG was mainly directed to the viral proteins NP, VP40, VP35 and sGP. These results, together with the high seroprevalence rates and ELISA positivity on highly diluted sera, support the specificity of the observed IgG reactivity. They thus imply that IgG antibodies were generated in response to ZEBOV exposure of the individuals concerned. In order to rule out false-positive antibody responses, we further investigated ZEBOV-specific T cell memory responses in seven randomly selected IgG+ individuals. Despite inter-individual variability, we observed higher IFN-γ production in CD8+ T cells from IgG+ individuals than in negative controls, a finding indicative of memory T cell responses to ZEBOV. It is now well recognized that CD8 T cell responses to acute viral infection can be divided into three distinct phases [37]–[40]. Antigenic stimulation leads to massive clonal expansion of naïve CD8 T cells and to the acquisition of effector functions, including IFN-γ and TNF-α production and cytotoxic activity that render the cells capable of killing virus-infected cells. Once the infection is resolved, 90–95% of activated effector CD8 T cells die through apoptosis, while the remainder form a long-lived population of memory cells. Memory CD8 T cells have an enhanced capacity to control secondary antigen exposure, through more efficient proliferation, rapid acquisition of effector functions, IFN-γ and TNF-α production, and migration to peripheral sites of infection [41]. Long-term protective memory after initial antigen exposure has been shown in individuals vaccinated against the viruses causing smallpox, yellow fever, measles and polio [42]. Memory CD8 T cell responses can persist for up to 75 years after vaccination, thus providing lifelong protection [43]. Furthermore, a recent study showed that 90% of individuals vaccinated against smallpox still had vaccinia virus-specific IFN-γ-producing CD8 T cells, indicating that long-term protection is mediated by memory CD8 T cell responses [44], [45]. Highly effective protection of experimental rodents and non human primates against Ebola virus infection after various types of vaccination is associated with the generation of ZEBOV-specific IFN-γ-producing CD8 T cells and antibody responses [46]–[52]. Consequently, the detection of both ZEBOV-specific IgG and IFN-γ-producing CD8 T cell responses in all the healthy seropositive individuals tested here shows that these individuals must have been exposed to ZEBOV. Although EHF is not always associated with bleeding, other symptoms (abrupt-onset high fever, severe diarrhea and vomiting, breast and chest pain, etc.) are frequently severe and are therefore easily remembered. Furthermore, even if bleeding does not occur, severe forms of ZEBOV infections are associated with high viral load in other body fluids and, therefore, with high infectivity, inducing human-to-human transmission and secondary cases. Such events are unlikely to go undetected in Gabon. For these reasons, we believe most of the seropositive persons identified in our survey had probably had mild or asymptomatic infection, or were simply exposed to viral particles.

The similar rates of EBOV seropositivity in non epidemic regions and outbreak areas of Gabon, together with the small number of survivors from past outbreaks, rule out an important role of human-to-human transmission and rather suggest direct or indirect contact with infected animals. Moreover importantly, ZEBOV is mainly excreted in blood, diarrhea and vomit. ZEBOV is present in very large amounts in these fluids (more than several million virions per mL) during the acute phase of the disease. Although viral particles have been detected in saliva and sweat from acutely ill patients, human-human transmission by this route has not been documented. This putative transmission route would be even more unlikely in the case of mild or asymptomatic disease which is associated with a much lower viral load. Only three animal species, in addition to humans, have been shown to be naturally infected by ZEBOV. Chimpanzees and gorillas are unlikely candidates, as infection occurs only occasionally and death ensues rapidly [16], [23], [24]. Moreover, wild populations are small and live deep in the forest far from villages, while physical contacts with humans are rare and generally involve dead carcasses.

Fruit bat species (*Hypsignathus monstrosus*, *Epomops franqueti* and *Myonycteris torquata*) are naturally infected by ZEBOV, suggesting they may act as natural reservoirs [18], [19]. All three bat species have broad geographical ranges that are known to include the entire tropical forest regions of equatorial central Africa [53]. These bat populations are particularly abundant in the forested areas of Gabon, where ZEBOV-specific seroprevalence rates are high compared to Lakeland and Savannah areas, that themselves harbor other species of bats [53]. Moreover, these bats roost in massive numbers on trees and consume their fruits, especially within and around villages. Thus, it is possible that ZEBOV antigenic stimulation or aborted infection could occur when villagers handle and eat fruits which have been contaminated by bat saliva that may contain infectious virus, inactivated virus, or simple viral antigens. Brief contact with non infectious viral particles is sufficient to induce the type of specific immunity observed in our study. Indeed, CD4+ and CD8+ T cells are capable of activating and proliferating after a short encounter with an antigen, without the need for continued antigenic stimulation [54]. Gender, hunting activity and contact with animals did not appear to influence the risk of IgG seropositivity in our study. It is noteworthy that small children are less exposed to potentially contaminated fruit, and that ZEBOV seropositivity increased linearly with age during childhood. These routes of infection have been strongly implicated in pig infection by nipah virus and horse infection by hendra virus, both viruses belonging to the *Paramyxoviridae* family [55], [56], another family of Mononegavirales (enveloped single-stranded viruses with an RNA monosegmented genome of negative polarity and capsids with helical symmetry).

This study provides important insights into ZEBOV circulation, human exposure and pathogenicity, and outbreak occurrence. In particular, we found a strikingly high proportion of individuals living in forested areas of Gabon who had both specific humoral and cellular immunity to ZEBOV. The high frequency of ‘immune’ individuals with no disease or outbreak history raises questions as to the real pathogenicity of ZEBOV for humans in ‘natural’ conditions. Added to the lack of identifiable risk factors, this points to bats as the main source of human exposure, through handling and ingestion of contaminated fruits. Past outbreaks in which no animal source was identified may have been due to fruit bat activity close to the villages concerned. Rural populations living in forested regions of the central African forest block thus appear to be highly exposed to the virus. More and more viruses are being detected in bats, some of which are known to be pathogenic for humans.

## Materials and Methods

### Study Area and Population

The survey was conducted in Gabon, Central Africa. Nearly 80% of Gabon is covered by rain forest, which is subdivided into three different types, from North-East to South-West of the country [57]: the northern-eastern forest located near Cameroon and the Republic of Congo, the interior forest (characterized mainly by the presence of Okoume trees), and the mountain forest. Gabon also has a grassland region located between the coastline and mountain forest, as well as a dry savannah located mainly in the south-east, south-west and center, and a wide “Lakeland” and long coastline to the west (Figure 1). Gabon has a surface area of 267,667 km^2^ and is divided into nine administrative regions comprising a total of 2,048 villages, most of which are located along roads (Figure 1). Few villages have more than 300 inhabitants.

The current serosurvey was conducted by a multidisciplinary team, including a doctor from the Gabonese Ministry of Health, a nurse, an epidemiologist, a virologist, a veterinarian and laboratory technicians. It focused on rural villages with fewer than 300 inhabitants located in the nine administrative regions of Gabon. It took place during nine one-month field missions, between 2005 and 2008. The survey covered 220 randomly selected villages (Figure 1), representing 10.7% of all villages in Gabon. The traditional chiefs of each village were first informed of the survey, followed, with the chief's approval, by all interested villagers. All inhabitants aged ≥16 years were eligible for the study. The study was described orally, and volunteers gave their signed informed consent to be enrolled in the study and for their blood samples to be used for future research studies. Each participant answered an anonymous oral questionnaire that included questions on demographics, lifestyle, work, social activities, diet and past medical illness. Villages were randomly selected in each of the nine provinces. We then randomly selected around 10% of the villages in each province, regardless of their size (50 to 300 persons). We systematically excluded children, elderly persons (more than ∼65 years), persons who were not permanent residents in the villages, and those who had been permanent residents for less than a year. Some inhabitants were not present at the time of our visit, because of their daily activities. In view of all these factors, the number of persons who refused was quite low (around 15% of the eligible population), representing 12 to 97 persons per village. This is unlikely to have had a significant impact on the results. A free medical examination and basic medicines were proposed to all participants and non participants. The samples were obtained after the interview. A total of 4,349 persons were enrolled.

In addition, we conducted a specific field mission to enroll laboratory-confirmed survivors of the three Gabonese ZEBOV outbreaks. After three months of investigations throughout the outbreak areas of Ogooué Ivindo region between June and August 2007, 20 survivors of the 2001 Mekambo, 1996 Booué and 1996 Mayibout outbreaks were identified and enrolled.

Finally, another specific field mission was carried out to enroll children aged 15 years or less. In total, 395 children aged between 2 and 15 years were randomly selected in six villages located in the northern-eastern forest region (Figure 1). In addition to a free medical examination and basic medicines, all children in each village had blood smears for malaria diagnosis and blood typing in the field. The results were given the following day and antimalarial drugs were provided if appropriate.

The two study protocols were reviewed and approved together by the Gabonese Ministry of Health (authorization n°00093/MSP/SG/SGAQM). Written consent was obtained from the Health Director of each region, the traditional chiefs of each village, and all participants. The parents' written consent was obtained for participating children.

### Blood Collection

A total of 4,349 blood samples were collected, usually in the villages' local healthcare centers. Our team was located in the main town of each administrative region, and field laboratory facilities were set up interior the General Hospital. Blood samples were collected in the villages on a daily basis, into two 7-ml Vacutainer tubes containing EDTA (VWR International, France). The tubes were then transported to the field lab, and plasma was obtained by centrifugation each evening. Plasma samples were kept at −20°C until the end of the field mission, then transported to Centre International de Recherches Médicales de Franceville (CIRMF), Gabon, and stored at −80°C until use.

Peripheral blood mononuclear cells (PBMC) were collected from 200 randomly selected individuals from among the 4,349 participants, and from the 20 survivors (see above). PBMC were separated from whole blood by density gradient centrifugation on lymphocyte separation medium (Eurobio) at 2,300 rpm for 20 min at room temperature. PBMC were then washed with phosphate buffered saline (PBS)-2% fetal calf serum (FCS), and were cryopreserved in FCS containing 10% DMSO in dry nitrogen until being transported to CIRMF. PBMC were finally stored at CIRMF in liquid nitrogen until immunological analysis.

### ZEBOV-Specific IgG Detection

An IgG ELISA method was used as previously described [27], with antigens kindly provided by the Special Pathogens Branch, Centers for Disease Control (Atlanta, USA). Briefly, Maxisorp plates (Nunc, Denmark) were coated with ZEBOV antigens diluted 1∶1000 in PBS, overnight at +4°C. Control plates were coated with uninfected Vero cell culture antigens in the same conditions. Sera were diluted 1∶1600 in 5% non fat milk in PBS-Tween 20 (0.1%) and incubated in the wells overnight at +4°C. Binding was visualized by using a peroxidase-labeled antibody to human IgG (Sigma, France) and the TMB detector system (Dynex Technologies, France). Optical density was measured at 450 nm with an ELISA plate reader. For each sample, we calculated the corrected optical density (OD) as the optical density of the antigen-coated well minus the OD of the corresponding control well.

A panel of 104 sera from individuals who had never visited Africa was obtained from Marseille, France, and used as negative controls for cut-off calculation.

### Western Blot

ZEBOV antigens were kindly provided by Dr. V.E. Volchkov (Laboratoire P4 Jean Mérieux, Lyon, France). They were separated on 10% NuPAGE Bis-Tris acrylamide gel (Invitrogen, UK) and transferred to nitrocellulose membranes for 1 hour at 30 V. The membranes were blocked overnight at 4°C in PBS, 5% nonfat milk, and 0.1% Tween 20. The test sera were diluted 1∶100 in PBS, 2.5% milk, 0.1% Tween, and incubated with the membrane for 2 hours at room temperature. The membrane was then incubated with HRP-conjugated goat anti-human IgG (H+L) (P.A.R.I.S., France) diluted 1∶5000 in PBS 2.5% milk, 0.1% Tween 20. Bound antibodies were visualized with a chemiluminescent substrate, following the manufacturer's protocol (Pierce, France). Three washes in PBS 0.1% Tween 20 were performed after blocking and each incubation step.

### Virus Titration

The virus used in this study belonged to *Zaire ebolavirus* lineage B and was isolated from the plasma of a patient who died during the 2003 outbreak in RC [17]. The virus was isolated on confluent monolayers of Vero E6 cells in 25-cm^2^ plastic tissue culture flasks. The cells were maintained in Dulbecco's modified Eagle's medium (DMEM, Life Technologies, France) supplemented with 2% heat-inactivated FCS, penicillin 10 U/ml and streptomycin 10 µg/ml (Invitrogen) at +37°C with 5% CO_2_. The virus stock was prepared from the supernatant of infected Vero E6 cells after two passages (Vero E6+2).

The ZEBOV stock was titered using a modified conventional plaque assay [58]. Serial 10-fold dilutions of 250 µL of supernatant were incubated in DMEM-2% heat-inactivated FCS for 1 h at 37°C on Vero E6 cells grown to confluence in 6-well plastic tissue culture plates. Two milliliters of 1.6% carboxymethyl cellulose (BDH Laboratories, Poole, United Kingdom) in complete DMEM-2% FCS were then added to each well, and the plates were incubated at 37°C with 5% CO2 for 5 days. The cells were fixed with 4% formaldehyde (Sigma, Courtaboeuf, France) in PBS, washed, and permeabilized with 0.5% Triton X-100 (Sigma) in PBS. A mix of monoclonal antibodies specific for ZEBOV GP, NP, VP40 and VP35, kindly provided by Dr. S. Baize (Laboratoire P4 Jean Mérieux, Lyon, France), was then added overnight at +4°C. A peroxidase-conjugated goat anti-mouse antibody diluted 1∶1600 (Sigma) was then added for 1 h at 37°C, and foci of infected cells were revealed with diaminobenzidine (Sigma).

All infections were performed in BSL-4 conditions at CIRMF (glove box model). ZEBOV-infected supernatants were removed in BSL-4 conditions and heat-inactivated at 56°C for 40 min. Inactivated ZEBOV (iZEBOV) was stored at −80°C until use.

### PBMC Culture and Antigenic Stimulation

Cryopreserved PBMC were rapidly thawed in a 37°C water bath, washed twice and incubated overnight at 37°C in RPMI 1640 culture medium (Life technologies, UK) with 10% heat-inactivated FCS (full RPMI-10% FCS), 1% penicillin-streptomycin, 1% nonessential amino acids, and 1 M HEPES. PBMC were then washed in RPMI medium, adjusted to a density of 1×10^6^ cells/mL, and cultured in 24-well flat-bottom culture plates. Cells were immediately stimulated with inactivated ZEBV (iZEBOV) at a multiplicity of infection (MOI) of 1. Three samples containing enough PBMC were again stimulated with iZEBOV at MOI 1 on day 5 (Figure 4). Interleukin-2 (Becton Dickinson, France) was added to the cells on day 2 after stimulation with iZEBOV (PI) at a final concentration of 100 U/mL. Negative controls included cells stimulated with an equal volume of uninfected Vero E6 supernatant (mock), and positive controls included cells stimulated with phytohemagglutinin A (PHA, 3 µg/mL final).

### PBMC Phenotyping and Intracellular Cytokine Staining

PBMC from seven individuals were analyzed on days 2 and 3 after iZEBOV stimulation, and PBMC from three of these individuals were also analyzed on days 5 and 7. PBMC were incubated for 5 hours with 10 µg/mL Brefeldin A (Sigma) and 2 µM monensin (Sigma), then harvested and washed in culture medium. Approximately 0.5×10^6^ cells were labeled for 20 min at room temperature with monoclonal antibodies (Beckman-Coulter, Geneva, Switzerland): PC5-conjugated anti-human CD3, PC7-conjugated anti-CD4 and PC7-conjugated anti-CD8. Isotype controls consisted of cells labeled with FITC-conjugated mouse IgG1 and PE-conjugated IgG1 (Beckman-Coulter). Cells were then fixed and permeabilized with IntraPrep reagent (Beckman-Coulter) as recommended by the manufacturer. Permeabilized cells were labeled for 20 min at room temperature with FITC-conjugated anti-IFN-γ and PE-conjugated anti-TNF-α (Beckman-Coulter). Cells were then washed, resuspended in 2% FCS, and analyzed with an FC500 four-color flow cytometer (Beckman Coulter) and CXP software (Beckman Coulter).

### Statistical Methods

All statistical analyses were performed using STATA software version 10 (STATA Corporation, College Station, Texas, USA). Overall and subgroup-specific ZEBOV seroprevalence rates were estimated and potential differences between subgroups were evaluated by using binomial survey-adjusted chi-square tests. Significance was assumed at p ≤0.01.
