# Correction: High Prevalence of Both Humoral and Cellular Immunity to *Zaire ebolavirus* among Rural Populations in Gabon

**DOI:** 10.1371/annotation/9bc62f9e-8386-4e9b-951c-1eeba930a41c

**Published:** 2010-02-26

**Authors:** Pierre Becquart, Nadia Wauquier, Tanel Mahlakõiv, Dieudonné Nkoghe, Cindy Padilla, Marc Souris, Benjamin Ollomo, Jean-Paul Gonzalez, Xavier De Lamballerie, Mirdad Kazanji, Eric M. Leroy

The third author, Tanel Mahlakõiv, should be noted as contributing equally to this work along with the first and second authors, Pierre Becquart and Nadia Wauquier. 

